# Supplementary material for: Development and validation of a quantitative instrument for measuring temporal and social disorientation in the Covid-19 crisis
Source: PLoS One. 2022 Nov 17;17(11):e0264604. doi: 10.1371/journal.pone.0264604 (PMC9671314; doi:10.1371/journal.pone.0264604)
Supplement: S1 File — (PDF) [file pone.0264604.s001.pdf]

Objet **RE:Question à propos d'éthique pour des questionnaires quantitatives anonymes**
De CNRS Comite d'Ethique
À FERNANDEZ VELASCO Pablo
Date 2021-03-30 10:13

Bonjour,

Nous vous invitons à consulter la page de notre site "Ethique au CNRS" la cellule Réglementation et Bioéthique.

Bien cordialement.

COMETS I comité d'éthique du CNRS
1 place Aristide Briand – 92195 MEUDON
+33 1 45 07 57 03 - +33 6 60 04 12 62
comite.ethique@cnrs.fr I comite-ethique.cnrs.fr

De : FERNANDEZ VELASCO Pablo [pfernandezvelasco@cri.ens.fr]
Envoyé : jeudi 25 mars 2021 12:53
À : CNRS Comite d'Ethique
Objet : Question à propos d'éthique pour des questionnaires quantitatives anonymes

Bonjour,

Je développe un questionnaire quantitative anonyme pour un projet basé au CNRS, et je me demandais si je dois m'adresser au comité d'éthique du CNRS pour qu'ils approuvent le questionnaire, et si oui, qui dois-je contacter et quelle est la procédure? Merci d'avance.

Bien cordialement,

Pablo Fernández Velasco
--
Coordinator of the Disorientation Research Seminar
Institut Jean Nicod, Département d'études cognitives,
ENS, EHESS, CNRS, PSL,
29 rue d'Ulm 75005 Paris, France
