# Supplementary material for: Development and validation of a quantitative instrument for measuring temporal and social disorientation in the Covid-19 crisis
Source: PLoS One. 2022 Nov 17;17(11):e0264604. doi: 10.1371/journal.pone.0264604 (PMC9671314; doi:10.1371/journal.pone.0264604)
Supplement: S2 File — (PDF) [file pone.0264604.s002.pdf]

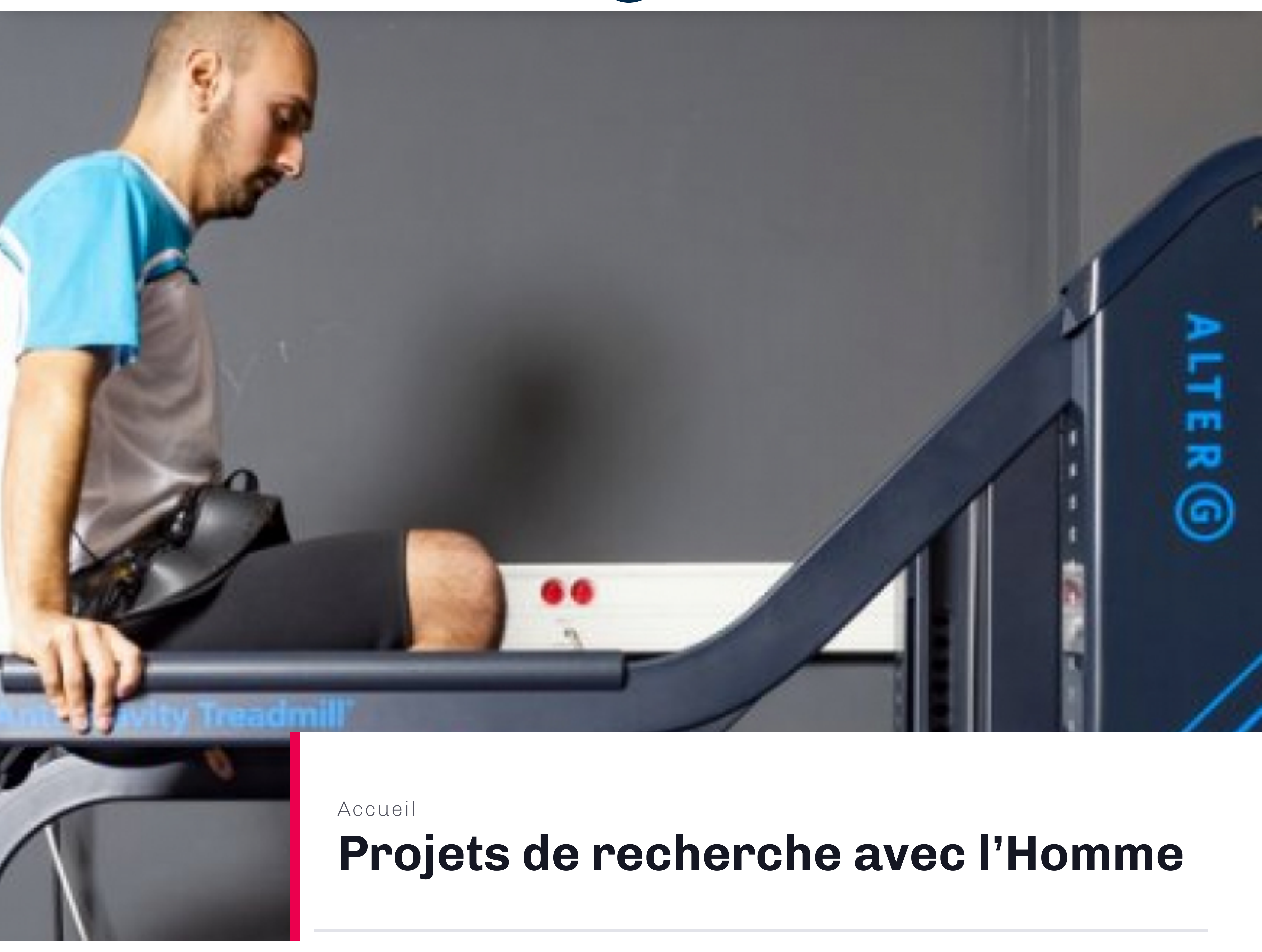

Projets de recherche avec l'Homme

19 mars 2021

Utiles dans de nombreux domaines scientifiques (biologie, sciences humaines, sciences de l'ingénierie…), les projets de recherche sur la personne, qui font intervenir des sujets volontaires ou des patients, leurs données ou leurs échantillons, posent des questions d'éthique particulières.

Vous trouverez ci-après les **principes éthiques et la réglementation à appliquer** en matière d'éthique **dans le cadre des différents types de recherche sur l'Homme**.

Les principes éthiques sont développés différemment selon les **conditions expérimentales qui président à la réalisation de la recherche** : le cadre législatif et les différents démarches décrites ci-dessous sont adaptés aux spécificités des situations rencontrées. Mais les activités de recherches sont toujours encadrées par des **principes éthiques** tels que la bienveillance, le respect de la dignité, de l'autonomie… Il s'agira également de **prévenir les risques biologiques éventuels sur la santé et l'environnement** découlant de la manipulation d'éléments biologiques.

Dans tous ces cas, l'information et le consentement du participant se prêtant à la recherche sont recommandés.

Quelles démarches pour quels projets de recherche ?

Pour savoir quelles démarches correspondent à un projet de recherche, reportez-vous :

1. à la **description ci-dessous** centrée sur la méthode expérimentale
   ET
2. à la Notice explicative sur les recherches sur l'Homme qui présente une clé de décision

1) Recherche avec utilisation de données personnelles, exploitations des données recueillies

Site de la CNIL

A. Données totalement anonymes : Pas de formalités obligatoires. Information et consentement du sujet recommandés.

B. Données personnelles même si non identifiantes (anonymisées, codifiées):

a. Exploitation manuelle
Information obligatoire des sujets qui peuvent s'y opposer.
Loi Informatique et Libertés

b. Exploitation informatisée
Loi Informatique et Libertés : Art. 7 , Chapitre IX et chapitre X, titre II

- Avis du Comité éthique et scientifique pour les recherches, les études et les évaluations dans le domaine de la santé (ministère de la Recherche)
- ET Autorisation de la Commission Nationale Informatique et Libertés (CNIL), par l'intermédiaire du Service DPD du CNRS (anciennement Correspondant Informatique et Libertés)

2) Recherche sur l'individu dans son ensemble

3) Recherche à partir d'éléments du corps humain

4) Recherche à partir d'embryons ou de cellules souches embryonnaires humaines

5) Recherche à partir de cellules souches pluripotentes induites humaines (iPS)
